# 6-Hydroxy­methyl-4-meth­oxy-2*H*-pyran-2-one (Opuntiol)

**DOI:** 10.1107/S1600536809053860

**Published:** 2009-12-19

**Authors:** Muhammad Athar Abbasi, Tayyaba Shahzadi, Mehmet Akkurt, Aziz-ur-Rehman, Tauheeda Riaz

**Affiliations:** a Department of Chemistry, Center for Natural Product Drug Development, Government College University, Lahore 54000, Pakistan; b Department of Physics, Faculty of Arts and Sciences, Erciyes University, 38039 Kayseri, Turkey

## Abstract

The title compound, C_7_H_8_O_4_, isolated from *Opuntia dillenii Haw* (Cacta­ceae), is almost planar [maximum deviation of 0.027 (2) Å] except for the H atoms of the methylene and methyl groups. The crystal packing is stabilized by C—H⋯O and O—H⋯O inter­molecular hydrogen bonds, resulting in the formation of a three-dimensional network.

## Related literature

For the use of the stem and fruit of *Opuntia dillenii Haw* (Cacta­ceae) in folk medicine, see: Chang *et al.* (2008 ▶). For phytochemical investigations of this plant, see: Qiu *et al.* (2002 ▶). For comparitive bond lengths, see: Allen *et al.* (1987 ▶).
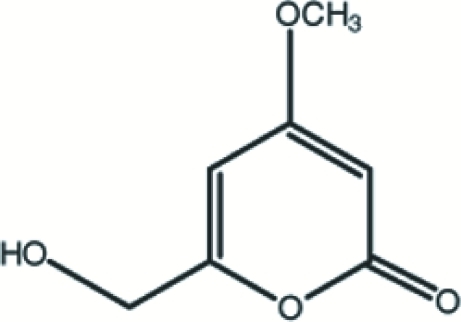


## Experimental

### 

#### Crystal data

C_7_H_8_O_4_
*M* *_r_* = 156.13
Monoclinic, 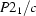

*a* = 4.0499 (5) Å
*b* = 18.101 (2) Å
*c* = 9.4743 (13) Å
β = 96.720 (7)°
*V* = 689.76 (15) Å^3^
*Z* = 4
Mo *K*α radiation
μ = 0.13 mm^−1^
*T* = 296 K
0.34 × 0.25 × 0.19 mm

#### Data collection

Bruker Kappa APEXII CCD area-detector diffractometer
6516 measured reflections
1268 independent reflections
882 reflections with *I* > 2σ(*I*)
*R*_int_ = 0.046

#### Refinement

*R*[*F* ^2^ > 2σ(*F* ^2^)] = 0.037
*wR*(*F* ^2^) = 0.094
*S* = 1.02
1268 reflections
105 parameters
1 restraint
H atoms treated by a mixture of independent and constrained refinement
Δρ_max_ = 0.16 e Å^−3^
Δρ_min_ = −0.16 e Å^−3^

### 

Data collection: *APEX2* (Bruker, 2007 ▶); cell refinement: *SAINT* (Bruker, 2007 ▶); data reduction: *SAINT*; program(s) used to solve structure: *SIR97* (Altomare *et al.*, 1999 ▶); program(s) used to refine structure: *SHELXL97* (Sheldrick, 2008 ▶); molecular graphics: *ORTEP-3* (Farrugia, 1997 ▶); software used to prepare material for publication: *WinGX* (Farrugia, 1999 ▶) and *PLATON* (Spek, 2009 ▶).

## Supplementary Material

Crystal structure: contains datablocks global, I. DOI: 10.1107/S1600536809053860/rk2184sup1.cif

Structure factors: contains datablocks I. DOI: 10.1107/S1600536809053860/rk2184Isup2.hkl

Additional supplementary materials:  crystallographic information; 3D view; checkCIF report

## Figures and Tables

**Table 1:** Hydrogen-bond geometry (Å, °)

*D*—H⋯*A*	*D*—H	H⋯*A*	*D*⋯*A*	*D*—H⋯*A*
O4—H1⋯O1^i^	0.836 (14)	2.47 (2)	3.1840 (19)	144.2 (17)
O4—H1⋯O2^i^	0.836 (14)	2.073 (13)	2.8678 (18)	158.6 (18)
C7—H7*B*⋯O4^ii^	0.96	2.58	3.494 (2)	159
C7—H7*C*⋯O2^iii^	0.96	2.57	3.504 (2)	164

Symmetry codes: (i) 
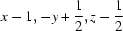
; (ii) 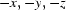; (iii) 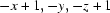.

**Fig. 1.**
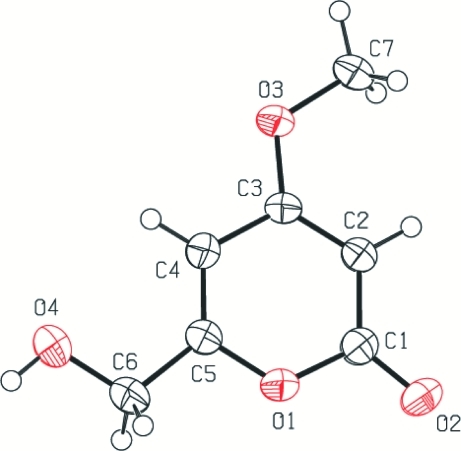
Molecular structure of the title compound with the atom numbering scheme. Displacement ellipsoids for non-H atoms are drawn at the 50% probability level. H atoms are presented as a small spheres of arbitrary radius.

**Fig. 2.**
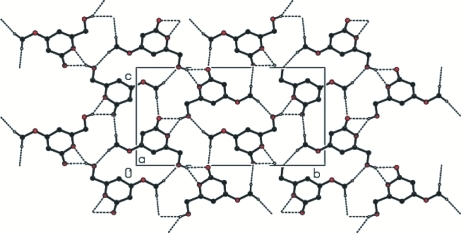
The packing and hydrogen bonding of the title compound viewed down a axis. Hydrogen atoms not involved in hydrogen bonding have been omitted for clarity.

**Fig. 3.**
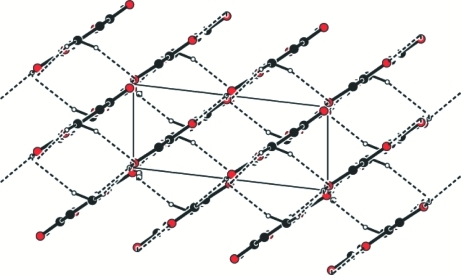
The packing and hydrogen bonding of the title compound viewed down b axis. Hydrogen atoms not involved in hydrogen bonding have been omitted for clarity.

## supplementary crystallographic information

### Comment

*Opuntia dillenii Haw* (Cactaceae) usually grows in semi-desert regions in the tropics and subtropics. The stem and fruit of this plant are used in a folk medicine for reducing cholesterol levels, treatment of gastric ulcers, inflammation, diabetes and several other diseases (Chang *et al.*, 2008). The phytochemical investigations on this plant have led to the isolation of various oxygenated constituents namely *opuntiol*, opuntioside-I, *p*-hydroxybenzoic acid, ethyl 3,4-dihydroxybenzoate, 3,4-dihydroxybenzoic acid, *L*-(-)-malic acid, (*E*)-ferulic acid, 4-ethoxy-6-hydroxymethyl-*α*-pyrone, 1-heptanecanol, vanillic acid, isorhamnetin, isorhamnetin-3-*O*-rutinoside, rutin, quercetin, 3,3'-dimethyl quercetin, 3-*O*-methyl quercetin, 3-*O*-methyl quercetin 7-*O*-*β*-*D*-glucopyranoside, kaempferol, kaempferol 7-*O*-*β*-*D*-glucopyranoside, kaempferol 7-*O*-*β*-*D*-glucopyranosyl-(1→4)- *β*-D-glucopyranoside, kaempferide, *β*-sitosterol, and manghaslin (Qiu *et al.*, 2002). In the present study we first time report the crystal structure of opuntiol using single-crystal XRD.

In the title molecule, I, shown in Fig. 1, bond lengths and angles display normal values (Allen *et al.*, 1987). Except the H atoms of the methlene and methyl groups, the molecule of I is almost planar, with maximum deviations of 0.027 (2)Å for O4, 0.023 (2)Å for C7, 0.020 (1)Å for O2 and -0.019 (2)Å for C2. For the methoxy and hydroxy methyl groups at the C3 and C5 positions in I, the C2–C3–O3–C7 and C4–C3–O3–C7 torsion angles are 0.9 (2)° and -178.94 (15)°, the O1–C5–C6–O4 and C4–C5–C6–O4 torsion angles are 177.77 (14)° and -2.9 (3)°, respectively.

In the crystal structure of I, there are non-classical C–H···O and classical O–H···O intermolecular hydrogen bonds (Table 1), forming a three-dimensional network (Figs. 2 and 3).

### Experimental

Plant Material:*Opuntia dillenii Haw* (whole plant) was collected from the areas of Mar Balochan, Sangla Hill, Distt. Nankana, Pakistan, in April 2008, and identified by Muhammad Ajaib (Taxonomist), Department of Botany, Government College University, Lahore.

Extraction and isolation: The shade-dried ground whole plant (7 kg) of *Opuntia dillenii Haw* was exhaustively extracted with methanol (10*L* ×4) at room temperature. The extract was evaporated to yield the residue (1.1 kg), which was dissolved in distilled water (2.0 *L*) and partitioned with *n*-hexane (2*L* ×4), chloroform (2*L* × 4), ethyl acetate (2*L* × 4) and *n*-butanol (2*L* × 4) respectively. The chloroform soluble extract (157 g) was subjected to column chromatography using hexane with gradient of CHCl_3_ and followed by methanol up to 100%. Fifteen fractions (Fr. 1-15) were collected. The Fr. 14 was loaded on flash silica gel and eluted with *Me*OH : CHCl_3_ (2 : 98) to get purified crystals of *opuntiol* (84.7 mg).

### Refinement

The H atom of the OH group was located in difference Fourier maps and were refined with a O–H distance restrained to 0.83 (1)Å, with displacement parameters fixed at 1.5 times *U*_eq_ of the parent O atom. The rest H atoms were placed geometrically, with C–H = 0.93-0.97 Å, and treated using a riding model, with *U*_iso_(H) = 1.2 or 1.5*U*_eq_(parent atom).

### Crystal data

**Table:**

C_7_H_8_O_4_	*F*(000) = 328
*M**_r_* = 156.13	*D*_x_ = 1.503 Mg m^−^^3^
Monoclinic, *P*2_1_/*c*	Mo *K*α radiation, λ = 0.71073 Å
Hall symbol: -P 2ybc	Cell parameters from 1306 reflections
*a* = 4.0499 (5) Å	θ = 2.3–23.4°
*b* = 18.101 (2) Å	µ = 0.13 mm^−^^1^
*c* = 9.4743 (13) Å	*T* = 296 K
β = 96.720 (7)°	Needle, colourless
*V* = 689.76 (15) Å^3^	0.34 × 0.25 × 0.19 mm
*Z* = 4	

### Data collection

**Table:**

Bruker Kappa APEXII CCD area-detector diffractometer	882 reflections with *I* > 2σ(*I*)
Radiation source: sealed tube	*R*_int_ = 0.046
graphite	θ_max_ = 25.5°, θ_min_ = 2.4°
φ– and ω–scans	*h* = −4→4
6516 measured reflections	*k* = −21→21
1268 independent reflections	*l* = −11→11

### Refinement

**Table:**

Refinement on *F*^2^	Secondary atom site location: difference Fourier map
Least-squares matrix: full	Hydrogen site location: inferred from neighbouring sites
*R*[*F*^2^ > 2σ(*F*^2^)] = 0.037	H atoms treated by a mixture of independent and constrained refinement
*wR*(*F*^2^) = 0.094	*w* = 1/[σ^2^(*F*_o_^2^) + (0.0406*P*)^2^ + 0.1053*P*] where *P* = (*F*_o_^2^ + 2*F*_c_^2^)/3
*S* = 1.01	(Δ/σ)_max_ < 0.001
1268 reflections	Δρ_max_ = 0.16 e Å^−^^3^
105 parameters	Δρ_min_ = −0.16 e Å^−^^3^
1 restraint	Extinction correction: *SHELXL97* (Sheldrick, 2008), FC^*^=KFC[1+0.001XFC^2^Λ^3^/SIN(2Θ)]^-1/4^
Primary atom site location: structure-invariant direct methods	Extinction coefficient: 0.0080 (19)

### Special details

**Table:**

Geometry. All s.u.'s (except the s.u. in the dihedral angle between two l.s. planes) are estimated using the full covariance matrix. The cell s.u.'s are taken into account individually in the estimation of s.u.'s in distances, angles and torsion angles; correlations between s.u.'s in cell parameters are only used when they are defined by crystal symmetry. An approximate (isotropic) treatment of cell s.u.'s is used for estimating s.u.'s involving l.s. planes.
Refinement. Refinement of *F*^2^ against ALL reflections. The weighted *R*-factor *wR* and goodness of fit *S* are based on *F*^2^, conventional *R*-factors *R* are based on *F*, with *F* set to zero for negative *F*^2^. The threshold expression of *F*^2^ > σ(*F*^2^) is used only for calculating *R*-factors(gt) *etc*. and is not relevant to the choice of reflections for refinement. *R*-factors based on *F*^2^ are statistically about twice as large as those based on *F*, and *R*-factors based on ALL data will be even larger.

### Fractional atomic coordinates and isotropic or equivalent isotropic displacement parameters (Å^2^)

**Table:**

	*x*	*y*	*z*	*U*_iso_*/*U*_eq_	
O1	0.6125 (3)	0.16542 (6)	0.30332 (12)	0.0376 (4)	
O2	0.9490 (3)	0.11978 (7)	0.48000 (14)	0.0495 (5)	
O3	0.3187 (3)	−0.03651 (6)	0.14748 (13)	0.0392 (4)	
O4	0.0594 (4)	0.22405 (7)	0.00555 (16)	0.0558 (5)	
C1	0.7497 (4)	0.10447 (9)	0.3778 (2)	0.0362 (6)	
C2	0.6491 (4)	0.03392 (9)	0.32627 (18)	0.0336 (6)	
C3	0.4283 (4)	0.02721 (9)	0.20778 (18)	0.0303 (5)	
C4	0.2914 (4)	0.09149 (9)	0.13544 (18)	0.0334 (6)	
C5	0.3878 (4)	0.15778 (9)	0.18521 (18)	0.0325 (6)	
C6	0.2807 (5)	0.23160 (9)	0.1300 (2)	0.0413 (6)	
C7	0.4484 (5)	−0.10405 (10)	0.2121 (2)	0.0437 (7)	
H1	−0.003 (6)	0.2662 (7)	−0.022 (2)	0.0840*	
H2	0.73440	−0.00810	0.37370	0.0400*	
H4	0.13750	0.08710	0.05500	0.0400*	
H6A	0.17300	0.25800	0.20100	0.0500*	
H6B	0.47320	0.25990	0.11000	0.0500*	
H7A	0.68640	−0.10410	0.21640	0.0660*	
H7B	0.35940	−0.14530	0.15650	0.0660*	
H7C	0.38600	−0.10780	0.30650	0.0660*	

### Atomic displacement parameters (Å^2^)

**Table:**

	*U*^11^	*U*^22^	*U*^33^	*U*^12^	*U*^13^	*U*^23^
O1	0.0434 (7)	0.0266 (7)	0.0405 (8)	−0.0032 (5)	−0.0045 (6)	−0.0040 (6)
O2	0.0590 (9)	0.0408 (8)	0.0437 (8)	−0.0065 (7)	−0.0149 (7)	−0.0057 (7)
O3	0.0503 (8)	0.0224 (7)	0.0420 (8)	−0.0014 (6)	−0.0073 (6)	−0.0020 (6)
O4	0.0688 (10)	0.0331 (8)	0.0596 (10)	0.0072 (7)	−0.0169 (8)	0.0056 (7)
C1	0.0385 (10)	0.0342 (11)	0.0347 (11)	−0.0020 (8)	−0.0002 (8)	0.0000 (9)
C2	0.0385 (10)	0.0256 (10)	0.0355 (11)	−0.0003 (8)	−0.0008 (8)	0.0019 (8)
C3	0.0333 (9)	0.0238 (9)	0.0335 (10)	−0.0023 (7)	0.0026 (8)	−0.0030 (8)
C4	0.0371 (10)	0.0312 (10)	0.0305 (10)	0.0010 (8)	−0.0021 (8)	0.0002 (8)
C5	0.0349 (10)	0.0274 (10)	0.0347 (10)	−0.0006 (8)	0.0022 (8)	0.0007 (8)
C6	0.0459 (11)	0.0267 (10)	0.0500 (12)	0.0014 (8)	0.0000 (9)	0.0015 (9)
C7	0.0548 (12)	0.0240 (10)	0.0501 (13)	0.0006 (8)	−0.0028 (10)	0.0008 (9)

### Geometric parameters (Å, °)

**Table:**

O1—C1	1.390 (2)	C4—C5	1.331 (2)
O1—C5	1.364 (2)	C5—C6	1.481 (2)
O2—C1	1.218 (2)	C2—H2	0.9300
O3—C3	1.340 (2)	C4—H4	0.9300
O3—C7	1.439 (2)	C6—H6A	0.9700
O4—C6	1.402 (2)	C6—H6B	0.9700
O4—H1	0.836 (14)	C7—H7A	0.9600
C1—C2	1.410 (2)	C7—H7B	0.9600
C2—C3	1.356 (2)	C7—H7C	0.9600
C3—C4	1.429 (2)		
			
O1···O4^i^	3.1840 (19)	C2···H7C	2.7800
O2···O4^i^	2.8678 (18)	C2···H7A	2.7200
O2···C6^i^	3.258 (2)	C7···H2	2.5100
O3···C4^ii^	3.415 (2)	C7···H7A^vii^	3.0900
O4···O2^iii^	2.8678 (18)	C7···H6A^x^	2.9900
O4···O1^iii^	3.1840 (19)	C7···H6B^x^	2.9800
O1···H1^i^	2.47 (2)	H1···H6B^vii^	2.5900
O2···H2^iv^	2.6900	H1···O1^iii^	2.47 (2)
O2···H7C^v^	2.5700	H1···O2^iii^	2.073 (13)
O2···H1^i^	2.073 (13)	H1···C1^iii^	2.676 (15)
O3···H4^vi^	2.6700	H2···C7	2.5100
O4···H7B^vi^	2.5800	H2···H7A	2.2800
O4···H4	2.5400	H2···H7C	2.3300
O4···H6B^vii^	2.7500	H2···O2^iv^	2.6900
C1···C4^viii^	3.365 (2)	H4···O4	2.5400
C1···C5^viii^	3.470 (2)	H4···O3^vi^	2.6700
C2···C3^viii^	3.473 (2)	H6A···C7^ix^	2.9900
C2···C4^viii^	3.496 (2)	H6B···O4^viii^	2.7500
C3···C2^vii^	3.473 (2)	H6B···H1^viii^	2.5900
C4···C2^vii^	3.496 (2)	H6B···C7^ix^	2.9800
C4···C1^vii^	3.365 (2)	H6B···H7C^ix^	2.5700
C4···C7^ii^	3.580 (3)	H7A···C2	2.7200
C4···O3^ii^	3.415 (2)	H7A···C7^viii^	3.0900
C5···C1^vii^	3.470 (2)	H7A···H2	2.2800
C6···C7^ix^	3.451 (3)	H7B···O4^vi^	2.5800
C6···O2^iii^	3.258 (2)	H7C···C2	2.7800
C7···C6^x^	3.451 (3)	H7C···H2	2.3300
C7···C4^ii^	3.580 (3)	H7C···H6B^x^	2.5700
C1···H1^i^	2.676 (15)	H7C···O2^v^	2.5700
			
C1—O1—C5	121.64 (13)	C1—C2—H2	120.00
C3—O3—C7	117.64 (14)	C3—C2—H2	120.00
C6—O4—H1	108.3 (14)	C3—C4—H4	121.00
O1—C1—O2	114.29 (14)	C5—C4—H4	121.00
O1—C1—C2	117.44 (15)	O4—C6—H6A	110.00
O2—C1—C2	128.25 (16)	O4—C6—H6B	110.00
C1—C2—C3	120.23 (15)	C5—C6—H6A	110.00
O3—C3—C2	125.70 (15)	C5—C6—H6B	110.00
C2—C3—C4	120.35 (15)	H6A—C6—H6B	108.00
O3—C3—C4	113.95 (14)	O3—C7—H7A	109.00
C3—C4—C5	118.87 (15)	O3—C7—H7B	109.00
O1—C5—C4	121.46 (15)	O3—C7—H7C	109.00
C4—C5—C6	128.79 (16)	H7A—C7—H7B	109.00
O1—C5—C6	109.75 (14)	H7A—C7—H7C	109.00
O4—C6—C5	109.94 (14)	H7B—C7—H7C	109.00
			
C5—O1—C1—O2	179.27 (15)	C1—C2—C3—O3	178.79 (16)
C5—O1—C1—C2	0.3 (2)	C1—C2—C3—C4	−1.1 (3)
C1—O1—C5—C4	−0.4 (2)	O3—C3—C4—C5	−178.94 (15)
C1—O1—C5—C6	179.00 (15)	C2—C3—C4—C5	0.9 (2)
C7—O3—C3—C2	−0.9 (2)	C3—C4—C5—O1	−0.2 (2)
C7—O3—C3—C4	178.94 (15)	C3—C4—C5—C6	−179.49 (17)
O1—C1—C2—C3	0.4 (2)	O1—C5—C6—O4	177.77 (14)
O2—C1—C2—C3	−178.35 (18)	C4—C5—C6—O4	−2.9 (3)

Symmetry codes: (i) *x*+1, −*y*+1/2, *z*+1/2; (ii) −*x*+1, −*y*, −*z*; (iii) *x*−1, −*y*+1/2, *z*−1/2; (iv) −*x*+2, −*y*, −*z*+1; (v) −*x*+1, −*y*, −*z*+1; (vi) −*x*, −*y*, −*z*; (vii) *x*−1, *y*, *z*; (viii) *x*+1, *y*, *z*; (ix) −*x*+1, *y*+1/2, −*z*+1/2; (x) −*x*+1, *y*−1/2, −*z*+1/2.

### Hydrogen-bond geometry (Å, °)

**Table:**

*D*—H···*A*	*D*—H	H···*A*	*D*···*A*	*D*—H···*A*
O4—H1···O1^iii^	0.836 (14)	2.47 (2)	3.1840 (19)	144.2 (17)
O4—H1···O2^iii^	0.836 (14)	2.073 (13)	2.8678 (18)	158.6 (18)
C7—H7B···O4^vi^	0.96	2.58	3.494 (2)	159
C7—H7C···O2^v^	0.96	2.57	3.504 (2)	164

Symmetry codes: (iii) *x*−1, −*y*+1/2, *z*−1/2; (vi) −*x*, −*y*, −*z*; (v) −*x*+1, −*y*, −*z*+1.
